# Association Between Nocturnal Oxygen Desaturation and the Severity of Obstructive Sleep Apnea: A Retrospective Study

**DOI:** 10.7759/cureus.112754

**Published:** 2026-07-15

**Authors:** Smahane Hammou Amar, Soumia M Fdil, Dalal Zagaouch, Khalid Bouti, Sanaa Hammi

**Affiliations:** 1 Department of Pulmonology, Mohammed VI University Hospital, Tangier, MAR; 2 Department of Pulmonology, Mohammed VI University Hospital, Abdelmalek Essaadi University, Tangier, MAR

**Keywords:** apnea-hypopnea index, nocturnal hypoxemia, nocturnal oxygen desaturation, obstructive sleep apnea (osa), oxygen desaturation index

## Abstract

Introduction

Obstructive sleep apnea (OSA) is characterized by recurrent respiratory events and intermittent hypoxemia. This study examined the associations between the apnea-hypopnea index (AHI) and nocturnal oxygenation parameters.

Materials and methods

This retrospective single-center study included all consecutive eligible patients with OSA confirmed by nocturnal ventilatory polygraphy in the Department of Pulmonology at Mohammed VI University Hospital Center, Tangier, between 2023 and 2025. Recordings were obtained using the SOMNOtouch™ RESP device (SOMNOmedics GmbH, Randersacker, Germany) and analyzed with DOMINO light software (version 1.5.0), with automated analysis followed by systematic manual review and validation by an experienced pulmonologist. Respiratory events were scored according to the American Academy of Sleep Medicine (AASM) scoring criteria, version 2.5. Normality was assessed before inferential analyses. Pearson correlations were complemented by Spearman rank correlations, and Holm adjustment was applied across the four prespecified associations within each correlation family.

Results

The mean age was 57.06 ± 14.52 years, 55.8% were male, and the mean BMI was 33.26 ± 8.14 kg/m². The mean AHI was 24.69 ± 20.77 events/h. AHI was positively associated with oxygen desaturation index (ODI) (Pearson r = 0.893) and T90 (r = 0.699) and inversely associated with minimum nocturnal SpO₂ (r = -0.510) and mean nocturnal SpO₂ (r = -0.683); all Holm-adjusted p-values were <0.001. Spearman analyses were directionally concordant.

Conclusion

In this small retrospective single-center cohort, AHI was associated with several nocturnal oxygenation indices. These exploratory findings suggest related physiological patterns but do not establish independence, causality, prognostic value, or clinical utility beyond AHI. Larger prospective studies with multivariable and outcome-based analyses are warranted.

## Introduction

Obstructive sleep apnea (OSA) is a highly prevalent yet substantially underdiagnosed sleep-related breathing disorder and an important public health concern [1]. It is characterized by recurrent episodes of complete (apnea) or partial (hypopnea) upper-airway collapse during sleep, resulting in intermittent hypoxemia, recurrent arousals, and disruption of normal sleep architecture. These disturbances are associated with impaired quality of life and excessive daytime sleepiness [2-4].

Intermittent hypoxemia is a key physiological feature of OSA and has been implicated in sympathetic nervous system activation, oxidative stress, systemic inflammation, and endothelial dysfunction, mechanisms that may contribute to OSA-associated cardiovascular and metabolic complications [4,5].

The apnea-hypopnea index (AHI) remains the conventional metric for classifying OSA severity but primarily quantifies respiratory-event frequency and does not fully characterize the depth, duration, or cumulative extent of nocturnal oxygen disturbance. Oxygenation indices derived from polysomnography or ventilatory polygraphy, including the oxygen desaturation index (ODI), minimum nocturnal oxygen saturation (SpO₂ nadir), mean nocturnal SpO₂, and cumulative time spent with SpO₂ below 90% (T90), reflect different aspects of nocturnal oxygenation. Previous studies have reported associations between hypoxemia-based measures and adverse cardiovascular outcomes [6-9].

Patients with similar AHI values may exhibit different nocturnal oxygenation profiles because the physiological consequences of respiratory events vary according to event duration, baseline oxygenation, obesity, cardiopulmonary reserve, and other patient-specific characteristics. Oxygenation metrics may therefore provide additional descriptive information on the physiological expression of sleep-disordered breathing; however, their independent prognostic value and potential role in clinical risk stratification require validation in appropriately designed prospective studies [6-9].

OSA severity is conventionally classified according to AHI as mild (AHI 5≤ to 15> events/h), moderate (AHI 15≤ to 30> events/h), or severe (AHI ≥30 events/h) [10]. Accordingly, this exploratory, hypothesis-generating study aimed to characterize nocturnal oxygenation abnormalities and examine their associations with AHI in patients with OSA. We hypothesized that higher AHI values would be associated with higher oxygen desaturation index (ODI) and T90 values and with lower minimum and mean nocturnal SpO₂. These associations were examined to characterize the relationship between respiratory-event frequency and routinely available oxygenation indices, without inferring causality, independent prognostic value, or established clinical utility beyond AHI.

## Materials and methods

Study type

This retrospective, single-center, exploratory cross-sectional study included all consecutive eligible patients with obstructive sleep apnea (OSA) confirmed by nocturnal ventilatory polygraphy in the Department of Pulmonology at Mohammed VI University Hospital Center, Tangier, Morocco, between 2023 and 2025. The study was designed as an exploratory and hypothesis-generating analysis.

Study population

Patients were eligible if they had OSA confirmed by nocturnal ventilatory polygraphy, defined as an apnea-hypopnea index (AHI) ≥5 events/hour, and had complete clinical, demographic, polygraphic, and nocturnal oxygenation data derived from recordings of sufficient technical quality. Patients were excluded in cases of incomplete medical records; predominance of central sleep apnea; decompensated chronic respiratory disorders likely to independently affect nocturnal oxygenation; clinically decompensated heart failure likely to independently affect nocturnal oxygenation or ventilatory parameters; neuromuscular disorders; and any other condition likely to substantially influence nocturnal ventilatory parameters.

Nocturnal ventilatory polygraphy and study variables

Nocturnal ventilatory polygraphy recordings were obtained using the SOMNOtouch™ RESP device (SOMNOmedics GmbH, Randersacker, Germany) and analyzed with DOMINO light software, version 1.5.0. Automated analysis was followed by systematic manual review and validation by an experienced pulmonologist. Respiratory events were scored according to the American Academy of Sleep Medicine (AASM) scoring criteria, version 2.5.

The AHI was defined as the total number of apneas and hypopneas per hour of recording and was expressed as events/hour. Nocturnal oxygenation was characterized using four routinely available pulse oximetry-derived parameters. The oxygen desaturation index (ODI) was defined as the number of oxygen desaturation events per hour of recording and was expressed as events/hour. T90 was defined as the cumulative time, expressed in minutes, during which SpO₂ remained below 90%. Mean nocturnal SpO₂ was defined as the average of all oxygen saturation values recorded during the monitoring period. SpO₂ nadir was defined as the lowest oxygen saturation value recorded during the monitoring period.

Statistical analysis

Statistical analyses were performed using SPSS Statistics for Windows version 20.0 (IBM Inc., Armonk, New York), whereas power analyses were conducted using G*Power. Continuous variables were summarized as means and standard deviations or as medians and interquartile ranges (IQRs), as appropriate according to their distributions. Categorical variables were expressed as counts and percentages.

Distributional assumptions were assessed before inferential analyses using the Shapiro-Wilk test, complemented, where appropriate, by graphical inspection of variable distributions. Four associations were prespecified: AHI with ODI, T90, minimum nocturnal SpO₂, and mean nocturnal SpO₂.

Pearson product-moment correlation coefficients (r) were calculated to assess linear associations between AHI and each nocturnal oxygenation parameter. Given potential departures from normality and sensitivity to influential observations, Spearman rank correlation coefficients (ρ) were additionally calculated as complementary sensitivity analyses. Two-sided p-values and 95% confidence intervals (CIs) were reported for the correlation coefficients.

To account for multiple testing, Holm’s step-down procedure was applied separately across the four prespecified associations within each correlation family (Pearson and Spearman). Both unadjusted and Holm-adjusted p-values were reported. Statistical significance was defined as a two-sided Holm-adjusted p-value <0.05.

Given the modest sample size and exploratory nature of the study, no a priori power calculation was performed. The sample size was determined by the number of consecutive eligible patients available during the predefined study period. A post hoc power analysis was conducted using G*Power to contextualize the statistical power associated with the observed bivariate correlations. This analysis was based on the available sample size, a two-sided significance level of α = 0.05, and the observed Pearson correlation coefficients.

## Results

Study population characteristics

A total of 52 patients with confirmed OSA were included. Participants ranged in age from 16 to 91 years (mean 57.06 ± 14.52 years), and 55.8% (n = 29) were male. Mean body mass index (BMI) was 33.26 ± 8.14 kg/m². Cardiovascular diseases were the most frequently reported (61.5%, n = 32), followed by obesity (53.8%, n = 28), dyslipidemia (32.7%, n = 17), and endocrine disorders (26.9%, n = 14). The distribution of the main comorbidities is presented in Table 1.

**Table 1:** Demographic characteristics and comorbidities of patients with OSA (n = 52) Data are presented as mean ± standard deviation, n (%), or range, as appropriate. BMI - body mass index; OSA - obstructive sleep apnea

Characteristic	Value
Age, years	57.06 ± 14.52 (range, 16-91)
Male sex	29 (55.8%)
BMI, kg/m²	33.26 ± 8.14
Cardiovascular diseases	32 (61.5%)
Hypertension	20 (38.5%)
Cardiac arrhythmias	5 (9.6%)
Obesity	28 (53.8%)
Moderate obesity	13 (25.0%)
Severe obesity	10 (19.2%)
Morbid obesity	5 (9.6%)
Dyslipidemia	17 (32.7%)
Endocrine disorders	14 (26.9%)
Diabetes mellitus	11 (21.2%)
Thyroid disorders	3 (5.8%)
Psychiatric disorders	6 (11.5%)
Depression	5 (9.6%)
Anxiety disorder	1 (1.9%)
Smoking	6 (11.5%)
Asthma	4 (7.7%)

The most frequently reported clinical symptom was snoring (94.2%, n = 49), followed by witnessed nocturnal apneas and excessive daytime sleepiness (69.2%, n = 36 each), and fatigue (65.4%, n = 34) (Table 2).

**Table 2:** Clinical symptoms reported in patients with OSA (n = 52) OSA - obstructive sleep apnea

Clinical symptoms	n (%)
Snoring	49 (94.2)
Witnessed nocturnal apneas	36 (69.2)
Excessive daytime sleepiness	36 (69.2)
Fatigue	34 (65.4)

Respiratory and nocturnal oxygenation parameters

The mean AHI was 24.69 ± 20.77 events/hour. OSA severity was classified as mild in 42.3% of patients, moderate in 28.8%, and severe in 28.8%. The mean nocturnal SpO₂ was 94.06 ± 4.02%, the minimum nocturnal SpO₂ was 80.27 ± 12.61%, the mean ODI was 17.89 ± 19.32 events/hour, and the mean T90 was 52.12 ± 110.10 minutes. Because T90 showed marked right-skewness, it was additionally summarized by its median (5.0 minutes; IQR 1-53 minutes). Descriptive respiratory and nocturnal oxygenation parameters are summarized in Table 3.

**Table 3:** Respiratory and nocturnal oxygenation parameters in patients with OSA (n = 52) Data are presented as mean ± standard deviation unless otherwise specified. T90 is additionally presented as median (IQR) because of its skewed distribution OSA - obstructive sleep apnea

Parameter	Value
Apnea-hypopnea index (AHI), events/h	24.69 ± 20.77
Snoring index	165.93 ± 164.20
Mean nocturnal SpO₂, %	94.06 ± 4.08
SpO₂ nadir, %	80.27 ± 12.61
Oxygen Desaturation Index (ODI), events/h	17.89 ± 19.32
T90, min	52.12 ± 110.10
T90, median (IQR), min	5 (1-53)
Mild OSA, n (%)	22 (42.3)
Moderate OSA, n (%)	15 (28.8)
Severe OSA, n (%)	15 (28.8)

Normality assessment

Shapiro-Wilk tests demonstrated significant departures from normality for all continuous variables: AHI (W = 0.791, p < 0.001), ODI (W = 0.752, p < 0.001), mean nocturnal SpO₂ (W = 0.694, p < 0.001), minimum nocturnal SpO₂ (W = 0.755, p < 0.001), and T90 (W = 0.505, p < 0.001). The departure from normality was most pronounced for T90.

Correlations between AHI and nocturnal oxygenation parameters

AHI was positively correlated with ODI (Pearson r = 0.893; 95% CI (0.820; 0.938); Holm-adjusted p < 0.001) and T90 (r = 0.699; 95% CI (0.526; 0.816); Holm-adjusted p < 0.001). Negative correlations were observed with minimum nocturnal SpO₂ (r = -0.510; 95% CI (-0.688; -0.276); Holm-adjusted p < 0.001) and mean nocturnal SpO₂ (r = -0.683; 95% CI (-0.806; -0.504); Holm-adjusted p < 0.001). Spearman rank correlations showed associations in the same direction (ODI: ρ = 0.737; T90: ρ = 0.523; minimum SpO₂: ρ = -0.473; mean SpO₂: ρ = -0.589). All associations remained statistically significant after Holm correction (Table 4).

**Table 4:** Correlations between AHI and nocturnal oxygenation parameters (n = 52) Raw p-values correspond to two-sided correlation tests. Holm-adjusted p-values account for multiple testing across the four prespecified associations within each correlation family. All analyses were based on n = 52 observations AHI - apnea-hypopnea index; ODI - oxygen desaturation index; T90 - cumulative time spent with oxygen saturation below 90%; SpO₂ - peripheral oxygen saturation; CI - confidence interval

Parameter	Pearson’s r	95% CI for Pearson's r	Pearson p-value	Holm-adjusted Pearson p-value	Spearman’s ρ	95% CI for Spearman's ρ	Spearman p-value	Holm-adjusted Spearman p-value
ODI	0.893	(0.820; 0.938)	<0.001	<0.001	0.737	(0.580; 0.841)	<0.001	<0.001
T90	0.699	(0.526; 0.816)	<0.001	<0.001	0.523	(0.292; 0.697)	<0.001	<0.001
Minimum nocturnal SpO₂	-0.510	(-0.688; -0.276)	<0.001	<0.001	-0.473	(-0.660; -0.229)	<0.001	<0.001
Mean nocturnal SpO₂	-0.683	(-0.806; -0.504)	<0.001	<0.001	-0.589	(-0.742; -0.376)	<0.001	<0.001

Post hoc power analysis

Post hoc power analyses performed using G*Power indicated statistical power exceeding 95% for all primary associations and greater than 99% for the strongest observed correlations (AHI-ODI and AHI-T90).

## Discussion

The present exploratory study examined the relationships between AHI and several routinely available nocturnal oxygenation indices in patients with OSA. Overall, higher respiratory-event frequency was associated with lower mean and minimum nocturnal SpO₂ and with higher ODI and T90 values. These findings indicate that respiratory-event frequency and nocturnal oxygenation are closely related physiological dimensions of OSA. However, because of the retrospective cross-sectional design, these associations should be interpreted as descriptive rather than causal and do not establish independent prognostic significance.

The inverse associations between AHI and both mean and minimum nocturnal SpO₂ are physiologically plausible because recurrent upper-airway obstruction results in repeated disturbances of gas exchange and intermittent hypoxemia [1,11]. Nevertheless, the severity of oxygen desaturation depends not only on respiratory-event frequency but also on event duration, baseline oxygenation, obesity, cardiopulmonary reserve, sleep stage, body position, and other individual characteristics. Consequently, patients with similar AHI values may experience markedly different nocturnal oxygenation profiles. Our findings therefore support the concept that oxygenation indices describe different physiological aspects of OSA, although they should not be interpreted as demonstrating independent clinical or prognostic value beyond AHI [9,12,13].

The association between AHI and ODI deserves particular consideration. Because both indices are directly derived from recurrent respiratory events, a strong positive correlation is physiologically expected. Accordingly, the very strong association observed in our cohort may partly reflect collinearity between two closely related measures rather than completely independent physiological information. At the same time, the two metrics are not interchangeable because the occurrence and magnitude of oxygen desaturation vary according to event duration, baseline oxygen saturation, obesity, cardiopulmonary reserve, and other patient-specific characteristics. Therefore, although ODI may provide additional descriptive information regarding the frequency of oxygen desaturation accompanying respiratory events, further prospective studies using multivariable and outcome-based analyses are needed to determine whether it provides clinically meaningful information beyond AHI [9,11,12].

T90 reflects another dimension of nocturnal hypoxemia by quantifying the cumulative duration of oxygen saturation below 90%. In the present study, higher AHI values were associated with longer T90, suggesting that increasing respiratory-event frequency may coexist with greater cumulative hypoxemic exposure. However, interpretation of this finding requires caution. T90 showed the greatest departure from normality and marked right-skewness, indicating that a relatively small number of patients experienced disproportionately prolonged nocturnal hypoxemia. This statistical distribution supports the complementary use of Spearman rank correlations and suggests that T90 estimates may be particularly sensitive to extreme observations. Moreover, T90 is not specific to OSA because prolonged nocturnal hypoxemia may also result from obesity-related hypoventilation, impaired baseline oxygenation, or concomitant cardiopulmonary disease. Consequently, although T90 may characterize cumulative nocturnal hypoxemia, its clinical relevance beyond conventional OSA severity measures remains uncertain and requires further investigation.

Previous investigations have consistently demonstrated associations between hypoxemia-based metrics and adverse cardiovascular outcomes [6-8,14-16]. However, these studies differ substantially from the present work. Azarbarzin et al. demonstrated that sleep apnea-specific hypoxic burden independently predicted cardiovascular mortality and incident heart failure using large prospective cohorts and adjusted survival analyses [6,8]. Likewise, Trzepizur et al. reported associations between sleep apnea-specific hypoxic burden and cardiovascular events or all-cause mortality using longitudinal multivariable analyses [7]. Baumert et al. further showed that the composition of nocturnal hypoxemic burden predicted cardiovascular mortality in older community-dwelling men [16]. Similarly, Kendzerska et al. demonstrated associations between OSA severity and long-term cardiovascular events and mortality in a large historical cohort [14], whereas Oldenburg et al. reported that nocturnal hypoxemia was associated with increased mortality among patients with stable heart failure [15]. In contrast, our study was a small retrospective exploratory analysis based on unadjusted cross-sectional correlations rather than longitudinal outcome assessment. Therefore, our findings should be interpreted as describing physiological associations within this cohort rather than providing evidence regarding independent prognostic value or clinical risk stratification. A comparison of the present study and major studies on nocturnal hypoxemia in OSA can be seen in Table 5.

**Table 5:** Comparison of the present study with major studies on nocturnal hypoxemia in OSA AHI - apnea-hypopnea index; MACE - major adverse cardiovascular events; MrOS - Osteoporotic Fractures in Men Study; ODI - oxygen desaturation index; OSA - obstructive sleep apnea; SHHS - Sleep Heart Health Study; SpO₂ - peripheral oxygen saturation; T90 - time spent with oxygen saturation below 90%

Study	Design and population	Primary metric	Statistics	Main findings	Difference from present study
Azarbarzin et al. (2018) [6]	Prospective community cohorts (MrOS: n = 2743; SHHS: n = 5111)	Sleep apnea–specific hypoxic burden	Adjusted Cox regression	Independent predictor of cardiovascular mortality.	Large prospective cohorts with longitudinal outcomes and multivariable analyses.
Trzepizur et al. (2022) [7]	Prospective cohort (n = 5358)	Sleep apnea–specific hypoxic burden; T90	Latent class + adjusted Cox	Hypoxic burden and T90 independently predicted MACE and all-cause mortality; symptom subtypes were not independently associated.	Longitudinal prognostic study with multivariable adjustment.
Azarbarzin et al. (2020) [8]	Prospective community cohorts (SHHS: n = 4881; MrOS: n = 2653)	Sleep apnea–specific hypoxic burden	Adjusted Cox regression	Predicted incident heart failure independently of AHI.	Longitudinal clinical outcomes.
Baumert et al. (2018) [16]	Prospective MrOS cohort (n = 2840)	T90, ODI, hypoxemic burden composition	Survival analysis	T90 independently predicted cardiovascular mortality.	Population-based prognostic study.
Present study	Retrospective single-center exploratory study (n = 52)	ODI, T90, mean/minimum SpO₂	Pearson & Spearman (Holm correction)	Higher AHI associated with higher ODI/T90 and lower mean/minimum SpO₂; exploratory physiological associations only.	Cross-sectional exploratory design; no multivariable adjustment or prognostic outcomes.

Intermittent hypoxemia has been implicated in sympathetic nervous system activation, oxidative stress, systemic inflammation, and endothelial dysfunction [4,5,11]. These mechanisms provide biological plausibility for the observed associations between respiratory-event frequency and nocturnal oxygenation. Nevertheless, the present study did not evaluate cardiovascular outcomes, and the observed associations should not be interpreted as evidence that nocturnal hypoxemia directly caused cardiovascular disease in this cohort. The relatively high prevalence of cardiovascular comorbidities may instead reflect the advanced age of the participants, obesity, hypertension, referral bias, and other measured or unmeasured confounding factors.

Obesity is particularly relevant when interpreting the present findings. Excess adiposity is a well-established risk factor for OSA and contributes to upper-airway collapsibility, altered respiratory mechanics, and reduced functional residual capacity [17-20]. In our cohort, obesity was highly prevalent, and the mean BMI was within the obese range. Obesity may therefore have influenced both respiratory-event frequency and nocturnal oxygenation independently. Similar considerations apply to age, sex, baseline oxygenation, hypertension, and cardiovascular comorbidities, all of which may partly explain the observed associations. Because no multivariable adjustment was performed, the independent contribution of these potential confounders cannot be determined.

The absence of multivariable adjustment represents another important consideration. Because the analyses were limited to bivariate correlations, it cannot be determined whether the observed relationships are independent of age, sex, BMI, hypertension, cardiovascular comorbidities, baseline oxygenation, or other clinically relevant variables. Given the modest sample size (n = 52), multivariable regression would have increased the risk of unstable estimates and model overfitting. Accordingly, larger prospective studies should evaluate these associations using appropriately adjusted multivariable models.

Several strengths should nevertheless be acknowledged. All consecutive eligible patients meeting predefined inclusion criteria were included, respiratory events were scored according to AASM version 2.5 criteria, and all recordings underwent systematic manual validation by an experienced pulmonologist. Furthermore, several routinely available nocturnal oxygenation indices were evaluated simultaneously. The statistical analysis included formal assessment of normality, complementary Spearman sensitivity analyses, and Holm adjustment for multiple comparisons. Post hoc power analyses were additionally reported for descriptive purposes to contextualize the statistical power associated with the observed correlations.

Several limitations should also be acknowledged. First, the retrospective design precludes causal inference and remains susceptible to selection bias and residual confounding. Second, the single-center hospital-based setting may limit external validity. Third, the modest sample size (n = 52) restricted statistical precision, prevented robust subgroup and multivariable analyses, and reinforces the exploratory nature of the study. Although post hoc power analyses demonstrated high statistical power for the observed correlations, these analyses should be interpreted descriptively and do not compensate for the exploratory design, retrospective methodology, or limited sample size. Finally, ventilatory polygraphy does not directly assess sleep stages or total sleep time, and some oxygenation indices, particularly T90, may be influenced by determinants of hypoxemia unrelated to obstructive respiratory events.

## Conclusions

In this exploratory retrospective single-center study, higher AHI values were associated with lower mean and minimum nocturnal oxygen saturation and with higher ODI and T90 values, indicating that respiratory-event frequency and nocturnal oxygenation are closely related physiological dimensions of OSA. However, these unadjusted cross-sectional associations should be interpreted cautiously. Given the modest sample size, the absence of multivariable adjustment for potential confounders such as age, body mass index, sex, and comorbidities, and the retrospective observational design, the present findings do not establish causality, independent associations, prognostic significance, or clinical utility beyond conventional AHI assessment. Larger prospective multicenter studies using appropriately adjusted multivariable analyses and longitudinal clinical outcomes are needed to determine whether nocturnal oxygenation metrics provide independent physiological or prognostic information and whether they have a role in clinical decision-making beyond AHI.

## References

[1] Punjabi NM (2008). The epidemiology of adult obstructive sleep apnea. Proc Am Thorac Soc.

[2] Slowik JM, Sankari A, Collen JF (2026). Obstructive Sleep Apnea. https://www.ncbi.nlm.nih.gov/books/NBK459252/.

[3] Sankri-Tarbichi AG (2012). Obstructive sleep apnea-hypopnea syndrome: etiology and diagnosis. Avicenna J Med.

[4] Lévy P, Kohler M, McNicholas WT (2015). Obstructive sleep apnoea syndrome. Nat Rev Dis Primers.

[5] Lavie L (2015). Oxidative stress in obstructive sleep apnea and intermittent hypoxia - revisited - the bad ugly and good: implications to the heart and brain. Sleep Med Rev.

[6] Azarbarzin A, Sands SA, Stone KL (2019). The hypoxic burden of sleep apnoea predicts cardiovascular disease-related mortality: the Osteoporotic Fractures in Men Study and the Sleep Heart Health Study. Eur Heart J.

[7] Trzepizur W, Blanchard M, Ganem T (2022). Sleep apnea-specific hypoxic burden, symptom subtypes, and risk of cardiovascular events and all-cause mortality. Am J Respir Crit Care Med.

[8] Azarbarzin A, Sands SA, Taranto-Montemurro L (2020). The sleep apnea-specific hypoxic burden predicts incident heart failure. Chest.

[9] Malhotra A, Ayappa I, Ayas N (2021). Metrics of sleep apnea severity: beyond the apnea-hypopnea index. Sleep.

[10] (2010). Recommendations for clinical practice. Obstructive sleep apnea hypopnea syndrome in adults (Article in French). Rev Mal Respir.

[11] Dewan NA, Nieto FJ, Somers VK (2015). Intermittent hypoxemia and OSA: implications for comorbidities. Chest.

[12] Parekh A (2024). Hypoxic burden - definitions, pathophysiological concepts, methods of evaluation, and clinical relevance. Curr Opin Pulm Med.

[13] Labarca G, Vena D, Hu WH (2023). Sleep apnea physiological burdens and cardiovascular morbidity and mortality. Am J Respir Crit Care Med.

[14] Kendzerska T, Gershon AS, Hawker G, Leung RS, Tomlinson G (2014). Obstructive sleep apnea and risk of cardiovascular events and all-cause mortality: a decade-long historical cohort study. PLoS Med.

[15] Oldenburg O, Wellmann B, Buchholz A (2016). Nocturnal hypoxaemia is associated with increased mortality in stable heart failure patients. Eur Heart J.

[16] Baumert M, Immanuel SA, Stone KL (2020). Composition of nocturnal hypoxaemic burden and its prognostic value for cardiovascular mortality in older community-dwelling men. Eur Heart J.

[17] Destors M, Tamisier R, Galerneau LM, Lévy P, Pepin JL (2017). Pathophysiology of obstructive sleep apnea syndrome and its cardiometabolic consequences (Article in French). Presse Med.

[18] Schwartz AR, Patil SP, Laffan AM, Polotsky V, Schneider H, Smith PL (2008). Obesity and obstructive sleep apnea: pathogenic mechanisms and therapeutic approaches. Proc Am Thorac Soc.

[19] Young T, Skatrud J, Peppard PE (2004). Risk factors for obstructive sleep apnea in adults. JAMA.

[20] Peppard PE, Young T, Palta M, Dempsey J, Skatrud J (2000). Longitudinal study of moderate weight change and sleep-disordered breathing. JAMA.

